# “Setting up and Tailoring Early Intervention Teams in a Already Established Healthcare System: the Experience of the Greater Lyon”

**DOI:** 10.1192/j.eurpsy.2022.187

**Published:** 2022-09-01

**Authors:** F. Haesebaert, N. Franck, J. Haesebaert

**Affiliations:** 1 Lyon Neuroscience Research Center, Psy R2, Bron, France; 2 CH le Vinatier, Pôle Centre Rive Gauche, Sur-cl3r-peps, Bron, France; 3 CH le Vinatier, Pôle Centre Rive Gauche & Centre Ressource De Réhabilitation Psychosociale, BRON, France; 4 CNRS & Université Lyon 1, Umr 5229, BRON, France; 5 Hospices Civils de Lyon, Service Recherche Et Epidémiologie Cliniques, Pôle De Santé Publique, Lyon, France

**Keywords:** Health services research, early intervention, Implementation, First Episode Psychosis

## Abstract

The implementation of early intervention services (EIS) dedicated to first episode psychosis (FEP) remains a challenge in France. In 2016, the London School of Economics published a report in which France appeared as a poorly developed country in terms of early intervention services. Since the 1980s, the French psychiatric “sectorization” system offers access to general psychiatric care with a graduated intensity (outpatient consultations, day hospitalization, full time hospitalization) targeting territories (“sectors”) of approximately 70,000 inhabitants. While this system has advantages in terms of universal access to care, it leaves little room for specialized services. The Greater Lyon agglomeration (2.4 million inhabitants) is composed of several psychiatric sectors administered by 3 psychiatric hospitals and a psychiatric emergency system administered by a University Hospital. Since 2018, various hospital and university stakeholders, patient associations and international partners, have been working together to tailor, set up and organize a care system for FEP patients. We present here how we have federated workforce resources initially working within 11 general psychiatric “sectors” and covering a population of 850,000 inhabitants. A 3-step process of (1) field analysis, (2) the creation of a community of practice composed of healthcare workers, researchers and service users, and (3) confrontation of the already existing healthcare with the logic of “principal components” for FEP care, allowed the creation of the outpatient “PEPS” service, which now offers continuous case management, a functional recovery program and new pathways to care to more than 200 patients with a diagnosis of first episode psychosis.

**Disclosure:**

No significant relationships.

